# Nrf2 promotes esophageal squamous cell carcinoma (ESCC) resistance to radiotherapy through the CaMKIIα-associated activation of autophagy

**DOI:** 10.1186/s13578-020-00456-6

**Published:** 2020-07-30

**Authors:** Di Xia, Xiao-Ran Zhang, Yan-Li Ma, Zhi-Jun Zhao, Ren Zhao, Yan-Yang Wang

**Affiliations:** 1 Graduate School, Ningxia Medical University, Yinchuan, 750004 Ningxia China; 2 Cancer Center, Union Hospital, Tongji Medical College, Huazhong University of Science and Technology, Wuhan, 430023 Hubei China; 3 Dept. of Laboratory Medicine, General Hospital of Ningxia Medical University, Yinchuan, 750004 Ningxia China; 4 Dept. of Radiation Oncology, General Hospital of Ningxia Medical University, Yinchuan, 750004 Ningxia China; 5 Cancer Institute, Ningxia Medical University, Yinchuan, 750004 Ningxia China

**Keywords:** Nrf2, CaMKIIα, Autophagy, Radiation resistance, ESCC

## Abstract

**Background:**

NF-E2-related factor 2 (Nrf2) is involved in the radiation resistance of esophageal squamous cell carcinoma (ESCC), but the underlying molecular mechanism is unclear. The purpose of our study was to explore the role of Nrf2 in the radiation resistance of ESCC and the potential molecular mechanism.

**Results:**

Nrf2 expression was introduced into Ec109 and KYSE-30 ESCC cells with lentivirus. CCK-8 and colony formation assays were used to evaluate the effect of Nrf2 on radioresistance in culture. The autophagy level was assessed by western blotting, flow cytometry, and confocal fluorescence microscopy. The effect of Nrf2 on the transcription of Ca2 +/calmodulin-dependent protein kinase II α (CaMKIIα) was studied by chromatin immunoprecipitation. We found that the overexpression of Nrf2 increased the radiation resistance of ESCC cells. Mechanistically, Nrf2 triggered the radiation resistance of ESCC cells by targeting CaMKIIα and subsequently activating autophagy. In addition, we found that Nrf2 directly regulated the transcription of CaMKIIα by binding to its promoter region. The effect of Nrf2 on radiation resistance was also explored in both a xenograft mouse model and ESCC patient samples. Consistent with the results of the in vitro study, high Nrf2 expression level resulted in in vivo radioresistance in an Ec109-derived xenograft mouse model. Furthermore, we also demonstrated that upregulations of both Nrf2 and CaMKIIα was closely related to lower survival rates of ESCC patients.

**Conclusions:**

Our study reveals that Nrf2 promotes the radiation resistance of ESCC by targeting CaMKIIα and subsequently activating autophagy, which is characterized by the suppression of phosphorylated mTOR and p62, activation of Beclin 1, and transformation of LC3-I to LC3-II.

## Background

NF-E2-related factor 2 (Nrf2) is a transcription factor that is critical for maintaining normal cellular biological processes [1–3]. Dysregulation of Nrf2 has been identified as one of the key molecular events in carcinogenesis [4–6]. In recent years, a number of studies have reported that the aberrant activation of Nrf2 enhances the resistance of esophageal squamous cell carcinoma (ESCC) to chemoradiotherapy (CRT) [7–9]. However, the exact molecular mechanism of Nrf2-mediated radiation resistance is not clear.

Autophagy is a biological process that involves the self-degradation of cytoplasmic macromolecules and organelles under conditions of nutritional stress. Autophagy can be activated by a variety of cancer treatments as an adaptive response to facilitate cancer survival [10, 11]. Recent studies have shown that autophagy can mediate the radiation resistance of ESCC. Zhu et al. [12] reported that autophagy activation played a key role in ESCC radiation resistance induced by eukaryotic extension factor 2 kinase (eEF2K). Ma et al. [13] showed that high mobility group box 1 (HMGB1) enhanced the radiation resistance of ESCC by activating autophagy. It was also found that increased autophagy was the main mechanism of ESCC radioresistance induced by liver kinase B1 (LKB1) [14]. The mechanism of autophagy-induced radiation resistance, especially whether autophagy is involved in Nrf2-mediated radiation resistance, is worthy of further study.

Ca2 +/calmodulin-dependent protein kinase II (CaMKII) is an important member of the calcium/calmodulin-activated protein kinase family, and CaMKII functions in synaptic stimulation and T cell receptor signal transduction [15]. An increasing number of studies have also shown that aberrant activation of CaMKII is the signal transduction node that promotes a variety of cancers [16]. In a previous study, we confirmed that CaMKIIα could affect the sensitivity of esophageal cancer cells to CDDO-Me, an Nrf2 inducer, by regulating the autophagy signaling pathway [17]. However, to date, there have been no reports on the role of CaMKIIα in Nrf2-mediated radiation resistance and autophagy activation.

In this study, we examined the potential mechanism of Nrf2-mediated radiation resistance in ESCC. Our study demonstrated that Nrf2 triggered radiation resistance by upregulating the level of autophagy level in ESCC cells. We also found that CaMKIIα was the key molecule involved in the autophagy activation and radiation resistance induced by Nrf2. Mechanistically, Nrf2 induced the expression of CaMKIIα by activating its transcriptional activity in ESCC. These results provide a new idea for further studies of the molecular mechanism of Nrf2-mediated radioresistance in ESCC and provide a potential means by which improve the radiosensitivity of ESCC by targeting the Nrf2/CaMKIIα/autophagy signaling pathway.

## Results

### Nrf2 triggers radiation resistance in esophageal cancer cells

**Fig. 1:**
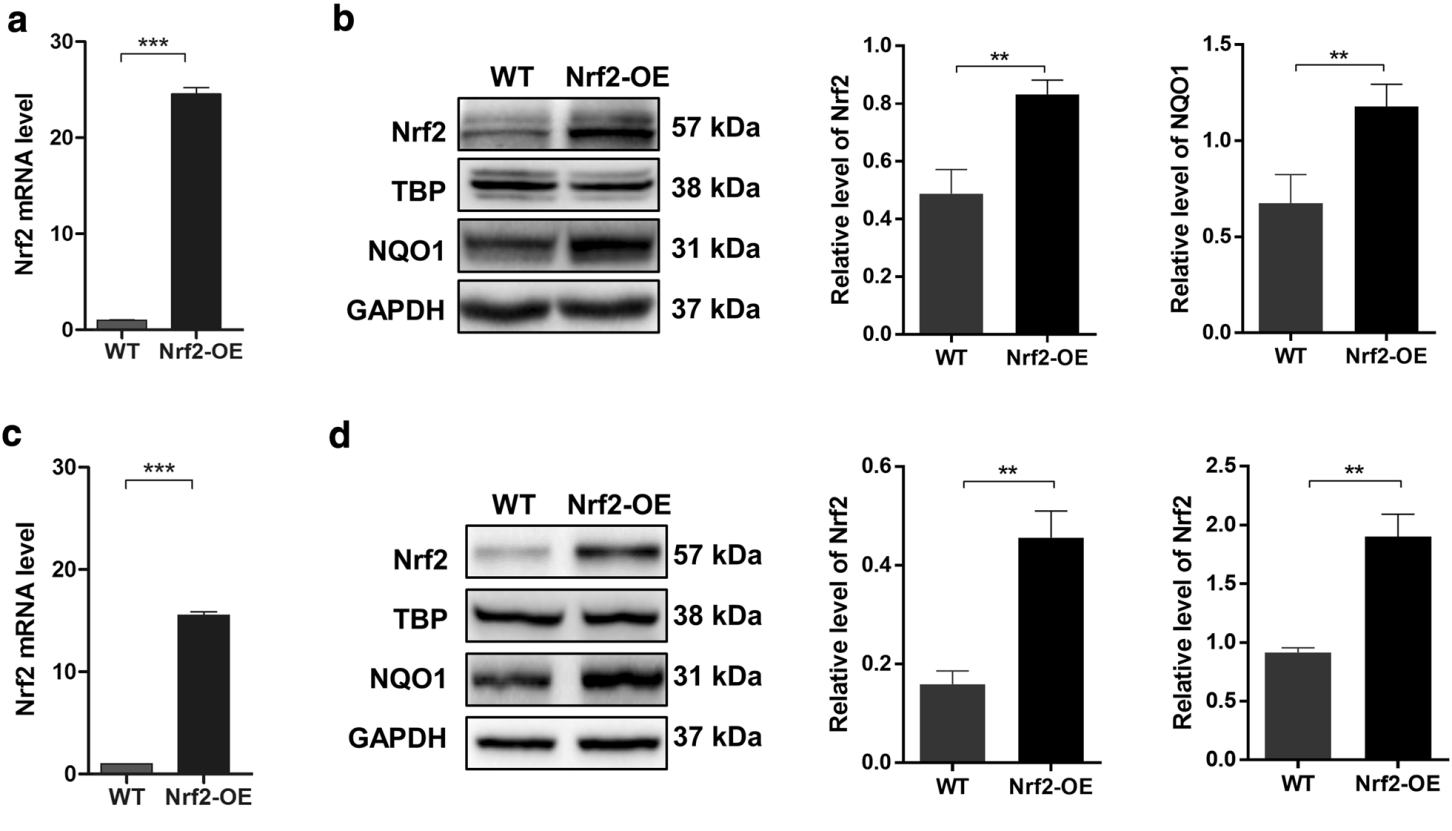
Induction of Nrf2 activity via transfection of lentivirus. The transfection effect of Nrf2 lentivirus was confirmed by qRT-PCR and western blot at mRNA and protein levels in Ec109 (**a**, **b**) and KYSE-30 (**c**, **d**) cells. The protein level of Nrf2 targeting gene NAD(P)H Quinone Dehydrogenase 1 (NQO1) was also evaluated by western blot in both Ec109 (**b**) and KYSE-30 (**d**) cells. All results are presented as the mean ± SEM from three repeated experiments. The differences in the two groups were obtained using Student’s *t* test. **P < 0.01 and ***P < 0.001

**Fig. 2:**
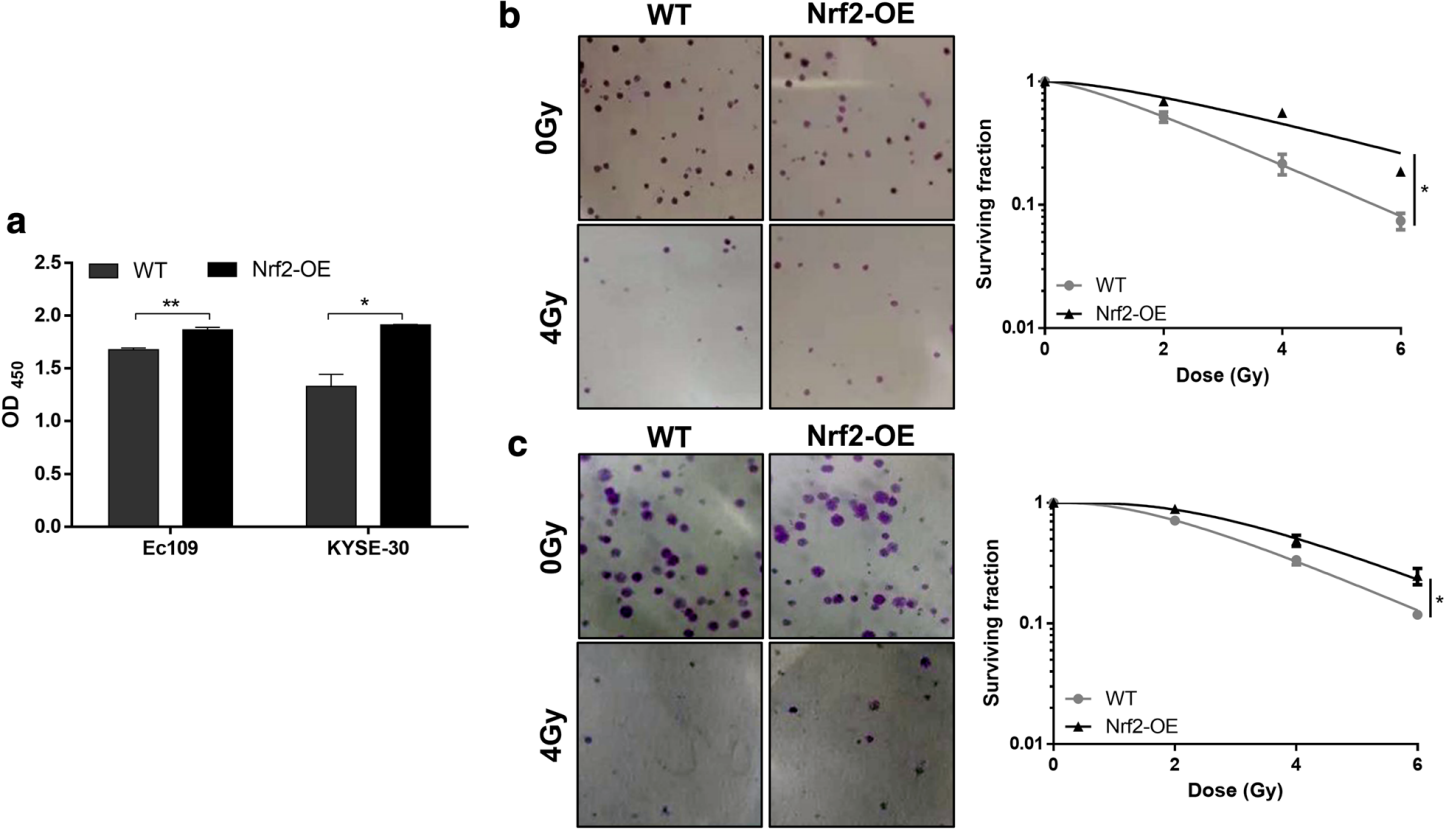
The overexpression of Nrf2 is related to the radiation resistance of ESCC cells. The radiosensitivity of wild-type (WT) and Nrf2-overexpressed (Nrf2-OE) Ec109 and KYSE-30 cells was detected by Cell counting kit-8 (CCK-8) assay (**a**) and colony formation test (**b** for Ec109 cells, **c** for KYSE-30 cells). Representative pictures of colony formation assay and cell survival curves (**b**, **c**) are shown. All results are presented as the mean ± SEM from three repeated experiments. The differences in the two groups were obtained using Student’s t-test. *P < 0.05 and **P < 0.01

To determine whether Nrf2 is involved in the radiation resistance of esophageal cancer cells, we first used a lentivirus transduction method to overexpress Nrf2 in both Ec109 and KYSE-30 cells. qRT-PCR and western blotting analyses confirmed that Nrf2 expression was elevated by lentivirus transduction in both Ec109 and KYSE-30 cells (Fig. 1). The upregulation of NAD(P)H quinone dehydrogenase 1 (NQO1) in ESCC cells was also observed by western blotting analysis, which indicated the induction of Nrf2 activity (Fig. 1b, d). In the subsequent analysis, ESCC cells were treated with various doses of irradiation. The effect of Nrf2 on the radiosensitivity of Ec109 and KYSE-30 cells was evaluated by CCK-8 and colony formation assays. Interestingly, Nrf2-overexpressing Ec109 cells were more resistant to radiation than wild-type cells, as demonstrated by both CCK-8 (Fig. 2a) and colony formation (Fig. 2b) assays. Similar results were obtained in KYSE-30 cells (Fig. 2a, c). These results indicate that Nrf2 triggers radiation resistance in esophageal cancer cells.

### Overexpression of Nrf2 increases autophagy in esophageal cancer cells

**Fig. 3:**
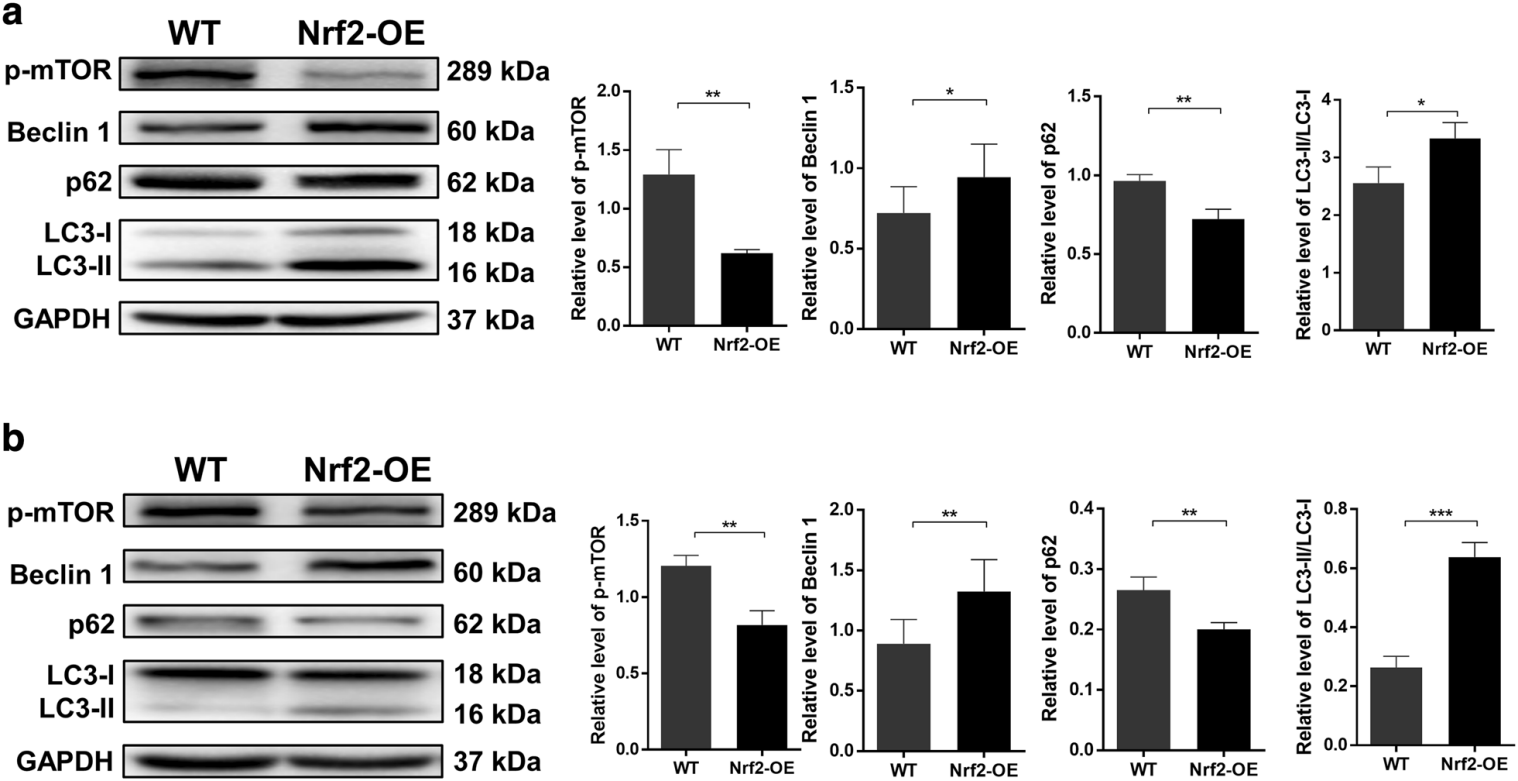
The overexpression of Nrf2 triggers the activation of autophagy in ESCC cells. The autophagy levels of wild-type (WT) and Nrf2-overexpressed (Nrf2-OE) ESCC cells were detected by western blotting. Representative western blotting images and the quantification and statistical analysis results of phosphorylated mTOR, Beclin 1, p62 and LC3-I/II in Ec109 cells (**a**) and KYSE-30 cells (**b**) are shown. The differences in the two groups were obtained using Student’s t-test. *P < 0.05, **P < 0.01, and ***P < 0.001

**Fig. 4:**
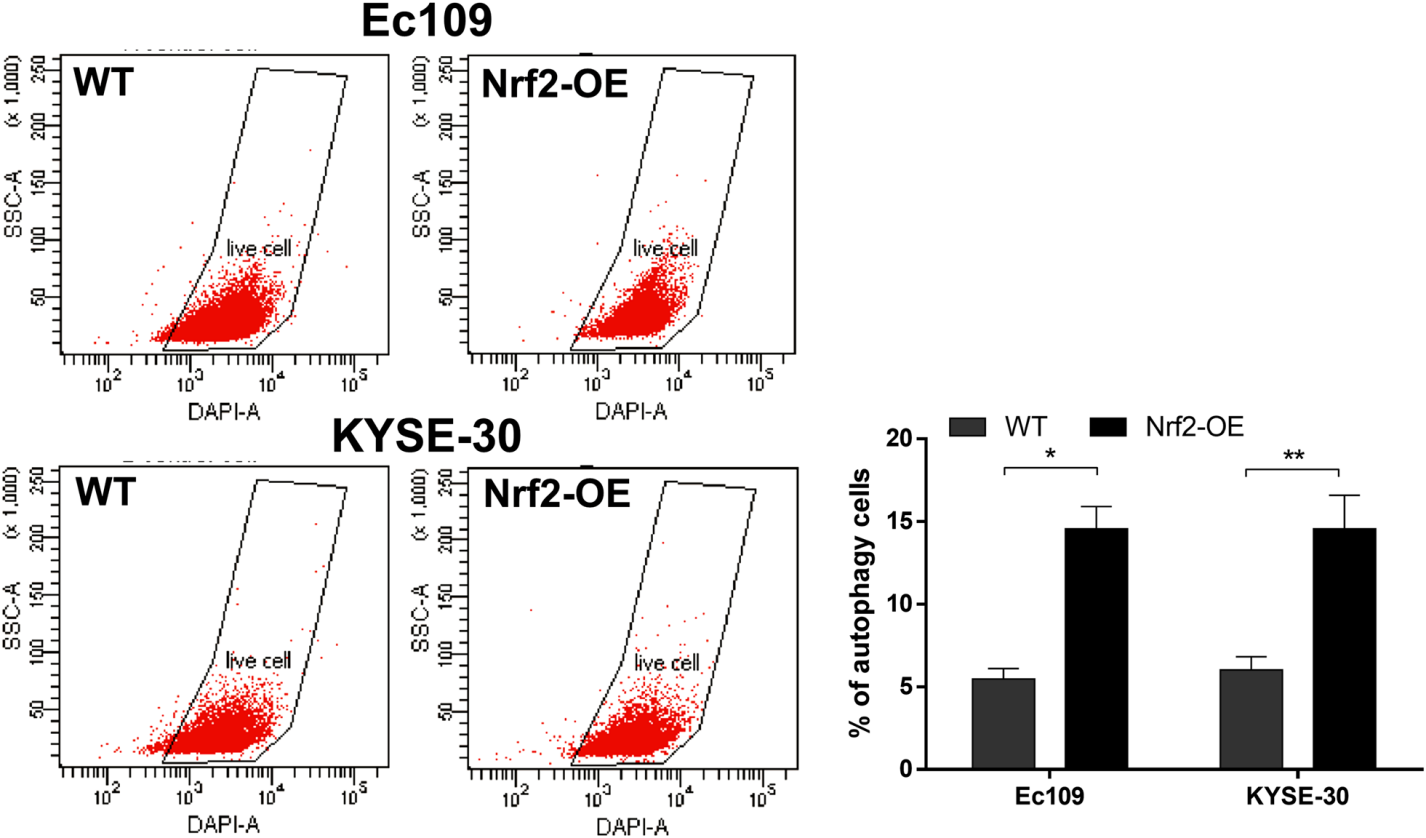
The overexpression of Nrf2 triggers the activation of autophagy in ESCC cells. The autophagy levels of wild-type (WT) and Nrf2-overexpressed (Nrf2-OE) ESCC cells were detected by flow cytometry. Representative images (left) and quantitative data (right) showed autophagy in Ec109 and KYSE-30 cells by flow cytometry analysis. The differences in the two groups were obtained using Student’s t-test. *P < 0.05 and **P < 0.01

**Fig. 5:**
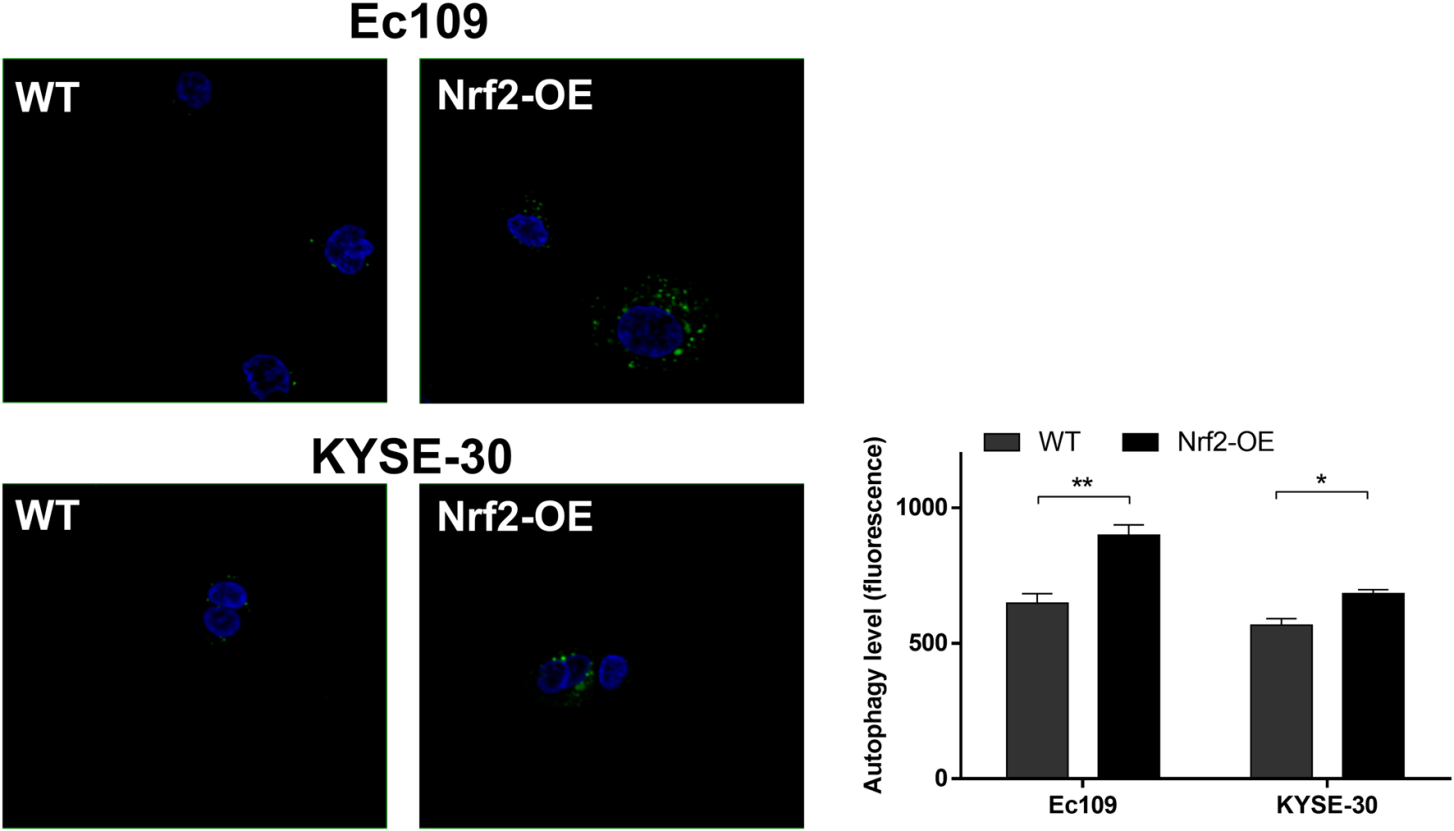
The overexpression of Nrf2 triggers the activation of autophagy in ESCC cells. The autophagy levels of wild-type (WT) and Nrf2-overexpressed (Nrf2-OE) ESCC cells were detected by confocal microscopy. Representative images (left) and quantitative data (right) showed the level of LC3 expression in Ec109 and KYSE-30 cells under confocal microscopy (green focus represents LC3, blue for DAPI). The differences in the two groups were obtained using Student’s t-test. *P < 0.05 and **P < 0.01

There is growing evidence that autophagy contributes to the radiation resistance of cancer cells. Decreased autophagy has been shown to make cancer cells more sensitive to radiotherapy [18–20]. To explore whether autophagy plays a role in the Nrf2-induced enhancement of radiation resistance, we transfected Nrf2 into Ec109 and KYSE-30 cells and then measured the levels of phosphorylated mTOR, Beclin 1, p62 and LC3. The western blotting results demonstrated that the levels of phosphorylated mTOR and p62 were decreased in Nrf2-overexpressing ESCC cells. Nrf2 triggered the upregulation of Beclin 1. The transformation of LC3-I to LC3-II in Nrf2-overexpressing Ec109 and KYSE-30 cells was also observed (Fig. 3). Next, we examined the autophagy-inducing effect of Nrf2 in ESCC cells by flow cytometry analysis. The results demonstrated that the number of autophagic Ec109 and KYSE-30 cells increased by threefold and 2.5-fold, respectively (Fig. 4). These results were further verified by confocal microscopy analysis. Confocal microscopy analysis showed that the green punctate structure of Nrf2-overexpressing cells was significantly increased, distributed in the perinuclear area and focally distributed in the cytoplasm, suggesting that the upregulation of Nrf2 increased the level of autophagy in esophageal cancer cells (Fig. 5). These data reveal that Nrf2 activates the autophagy flux in esophageal cancer cells, which may contribute to radiation resistance.

### Nrf2 promotes esophageal cancer cell radioresistance through the activation of autophagy via CaMKIIα

**Fig. 6:**
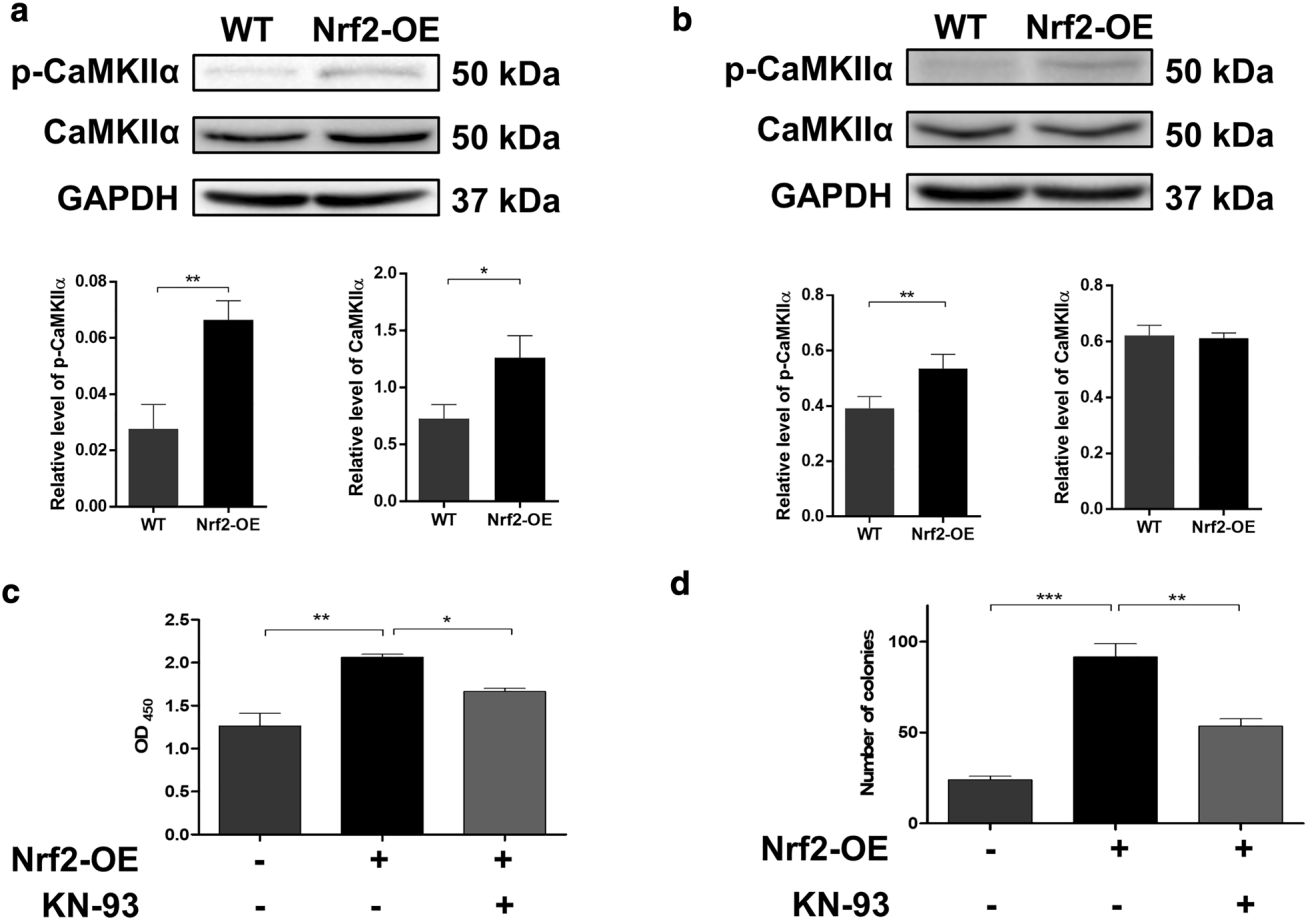
Nrf2 enhances radiation resistance by up-regulating the expression of CaMKIIα in ESCC cells. The expression levels of phosphorylated CaMKIIα and CaMKIIα in wild-type (WT) and Nrf2 overexpressed (Nrf2-OE) ESCC cells were detected by western blotting (**a** for Ec109 cells, **b** for KYSE-30 cells). Nrf2-OE Ec109 cells were pretreated with 15 μM KN-93 for 12 h and then irradiated with 4 Gy X-ray. After that, the radiation resistance was detected by Cell counting kit-8 (CCK-8) (**c**) and colony formation test (**d**). All results are presented as the mean ± SEM from three repeated experiments. The differences among the groups were obtained using Analysis of Variance (ANOVA). *P < 0.05, **P < 0.01, and ***P < 0.001

**Fig. 7:**
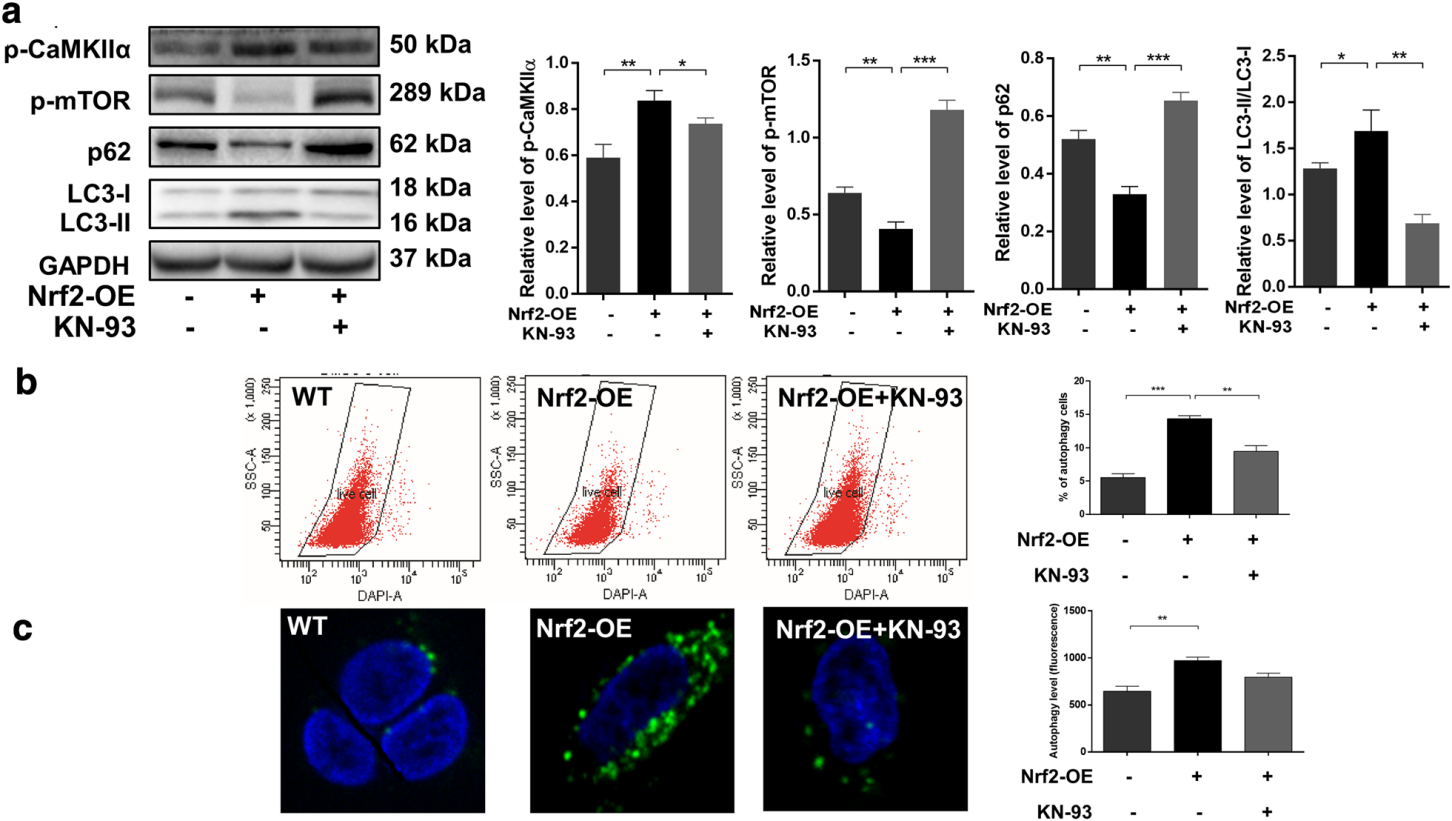
Nrf2-induced autophagy is partially rescued by pretreatment of CaMKIIα inhibitor. Nrf2-overexpressed (Nrf2-OE) Ec109 cells were pretreated with 15 μM KN-93 for 12 h, and then autophagy levels were analyzed by western blotting, flow cytometry and confocal microscopy, respectively. Representative western blotting images and the quantification and statistical analysis results of phosphorylated CaMKIIα, phosphorylated mTOR, p62 and LC3-I/II in Nrf2-OE Ec109 cells (**a**) are shown. Representative images and quantitative data showed autophagy in Nrf2-OE Ec109 cells by flow cytometry analysis. **b** Representative images and quantitative data show the expression level of LC3 in Nrf2-OE Ec109 cells under confocal microscope (green focus represents LC3, blue represents DAPI). **c** All results are presented as the mean ± SEM from three repeated experiments. The differences among the groups were obtained using Analysis of Variance (ANOVA). *P < 0.05, **P < 0.01, and ***P < 0.001

Our previous study reported that CaMKIIα could be upregulated by CDDO-Me in esophageal cancer cells [17]. To determine whether CaMKIIα is involved in the radiation resistance triggered by Nrf2, a western blotting assay was first performed. As shown in Fig. 6a, b, increased protein levels of both CaMKIIα and phosphorylated CaMKIIα were detected in Nrf2-overexpressing Ec109 cells. In KYSE-30 cells, only increased levels of phosphorylated CaMKIIα were observed. Next, we treated Nrf2-overexpressing Ec109 cells with the CaMKIIα inhibitor KN-93. The CCK-8 (Fig. 6c) and colony formation (Fig. 6d) assay results demonstrated that KN-93 treatment could partially reverse the radiation resistance triggered by Nrf2. To further verify the role of CaMKIIα in Nrf2-mediated radiation resistance, the autophagy levels of Nrf2-overexpressing Ec109 cells treated with KN-93 were evaluated by western blotting, flow cytometry and confocal microscopy. As expected, we found that Nrf2-induced autophagy was reversed by the CaMKIIα inhibitor (Fig. 7). Taken together, we found that Nrf2 enhanced the radioresistance of ESCC cells by targeting CaMKIIα and activating autophagy.

### Nrf2 activates CaMKIIα transcription by regulating its promoter activity

**Fig. 8:**
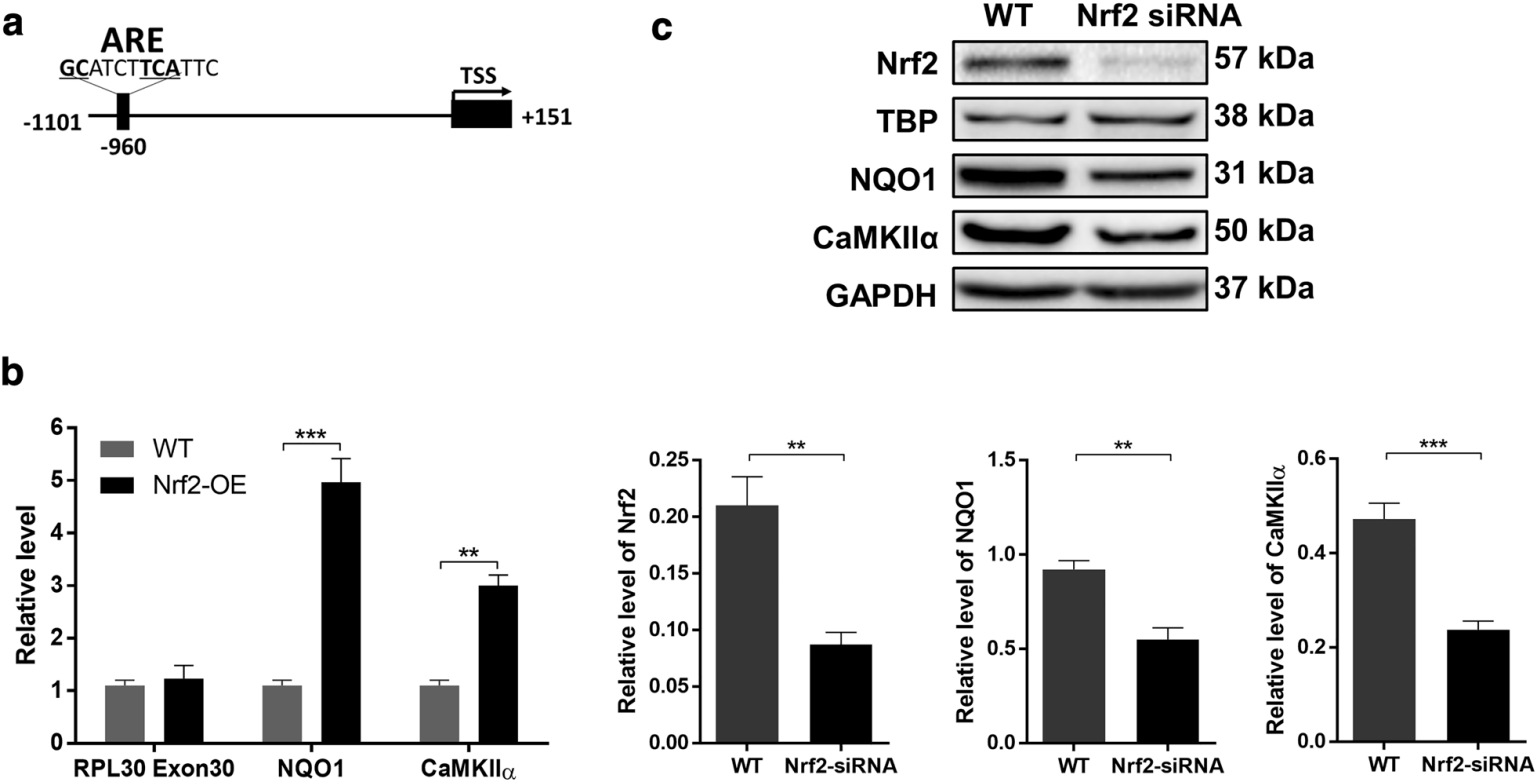
Nrf2 regulates CaMKIIα transcription through binding to its promoter. Schematic diagram of Nrf2-binding site (antioxidant response elements, ARE) which locates in − 960 bp of the CaMKIIα promoter. **a** Nrf2 ChIP followed by PCR amplification of the putative CaMKIIα ARE. Following Nrf2 ChIP, qPCR was done to measure the presence of CaMKIIα ARE, NQO1 ARE (positive control), and RPL30 Exon 3 (negative control). **b** Representative western blotting images and the quantification and statistical analysis results showed the level of NQO1 and CaMKIIα with or without Nrf2 knockdown. **c** All results are presented as the mean ± SEM from three repeated experiments. The differences in the two groups were obtained using Student’s t-test. **P < 0.01, and ***P < 0.001

Since Nrf2 is a transcription factor, we hypothesized that Nrf2 may directly regulate the transcription of CaMKIIα. To prove that Nrf2 affects the transcription of CaMKIIα by acting on its promoter, we first performed bioinformatics analysis. Online database screening (http:/mbs.cbrc.jp/research/db/TFSEARCH.html) showed that the Nrf2 binding site (antioxidant response element, ARE) was located at − 960 bp from the transcription initiation site of the CaMKIIα promoter region (Fig. 8a). To examine the interaction between Nrf2 and the CaMKIIα ARE, a chromatin immunoprecipitation (ChIP)-qPCR (ChIP-qPCR) assay was performed. The results showed that the qPCR signal specific to the CaMKIIα ARE was increased in Nrf2-overexpressing ESCC cells compared with wild-type ESCC cells. The same trend was observed in the canonical ARE located upstream of NQO1 but not in RPL30-exon 3, which is a non-Nrf2 target sequence (Fig. 8b). Finally, western blotting analysis results revealed that the level of CaMKIIα was significantly downregulated in the Nrf2-siRNA group (Fig. 8c). These results suggest that Nrf2 activates CaMKIIα transcription by regulating its promoter activity.

### Nrf2 enhances esophageal cancer radiation resistance in vivo

**Fig. 9:**
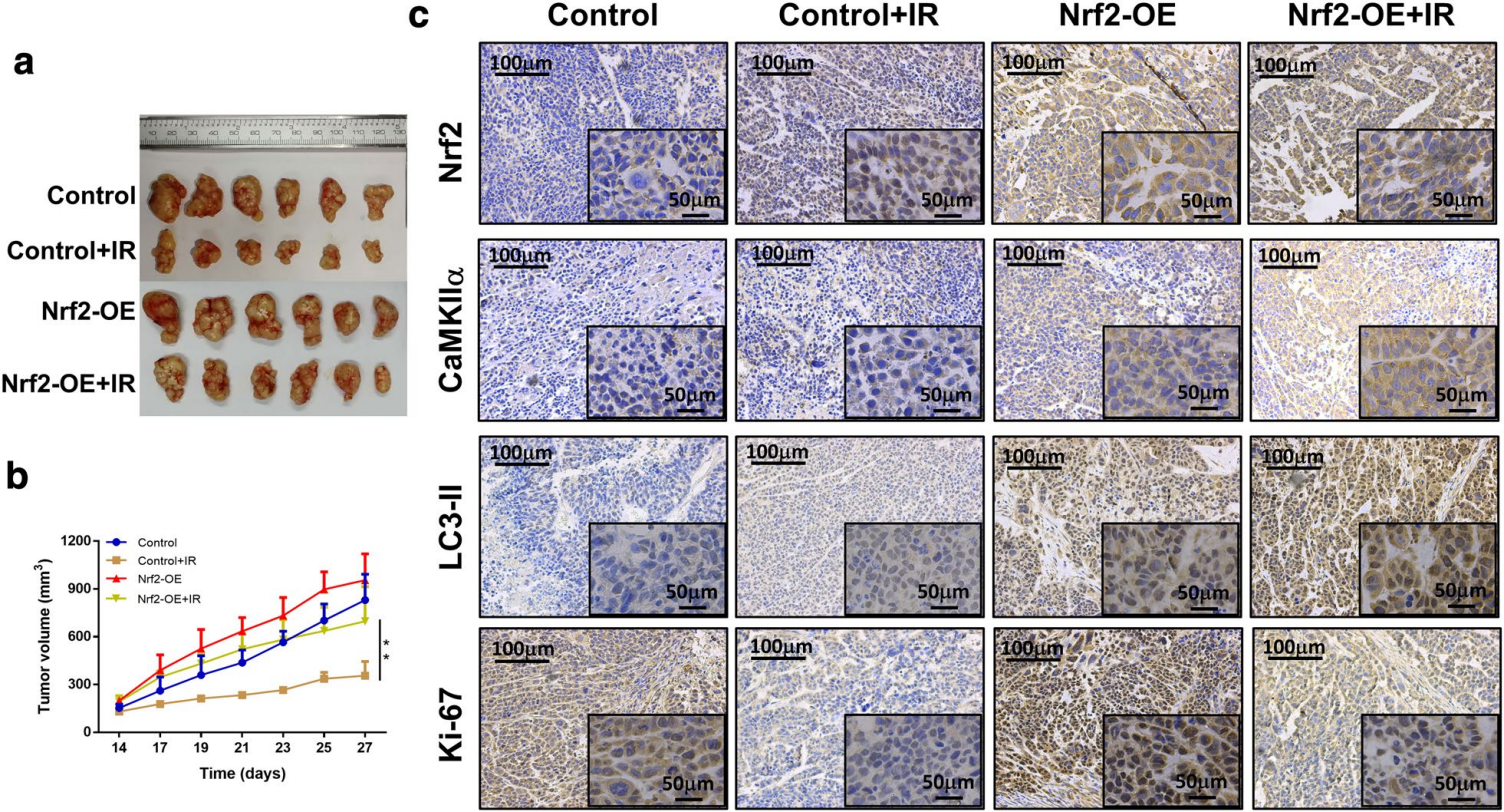
Overexpression of Nrf2 increases radiation resistance of Ec109 xenograft in nude mice. The growth images of wild-type (WT) and Nrf2-overexpressed (Nrf2-OE) Ec109 tumor in nude mice administrated with or without irradiation (IR, 6 Gy). **a** The growth curves of WT and Nrf2-OE Ec109 tumor in nude mice administrated with or without irradiation (IR, 6 Gy). **b** The expressions of Nrf2, CaMKIIα, LC3-II, and Ki-67 were examined by immunohistochemistry staining in the Ec109 tumor tissues of nude mice. **c** All results are presented as the mean ± SEM of 6 mice in each group. The differences among the groups were obtained using Analysis of Variance (ANOVA). **P < 0.01

To explore the function of Nrf2 in mediating ESCC radioresistance in vivo, we established an ESCC xenograft model by subcutaneous injection of wild-type Ec109 cells and Nrf2-overexpressing Ec109 cells, and we treated the tumor-bearing mice with irradiation. Radiotherapy inhibited the proliferation of both types of Ec109 cells in vivo. However, the overexpression of Nrf2 could largely eliminate the radiosensitivity of the Ec109 tumors (Fig. 9a, b). Consistent with the in vitro results, immunohistochemistry (IHC) staining showed that Nrf2 promoted the expression of CaMKIIα and LC3-II in vivo (Fig. 9c). Taken together, our observations show that Nrf2 enhances the radiation resistance of ESCC in vivo.

### Upregulations of the expression of both Nrf2 and CaMKIIα is related to the prognosis of esophageal cancer patients

**Fig. 10:**
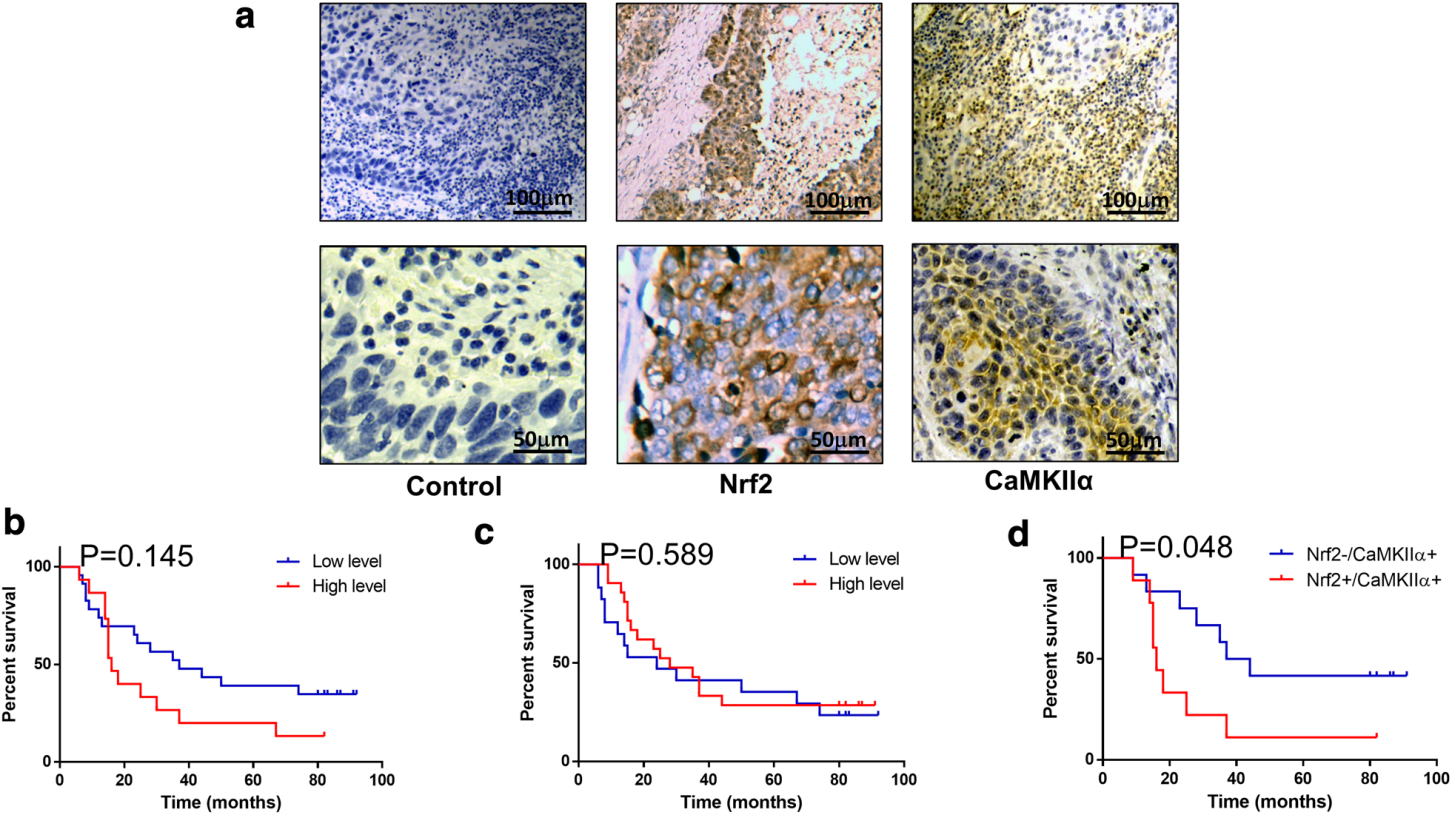
The expression of Nrf2 and CaMKIIα is associates with poor prognosis of patients with ESCC. Immunohistochemical method was used to detect the expression of Nrf2 and CaMKIIα in tumor tissues of patients with ESCC. **a** Effects of different levels of Nrf2 (**b**), CaMKIIα (**c**), and Nrf2 (+) + CaMKIIα (+) (**d**) on the overall survival of patients with ESCC. The overall survival time of ESCC patients with Nrf2 (+) + CaMKIIα (+) status was significantly shortened (P = 0.048)

To further explore the function of Nrf2 and CaMKIIα in esophageal cancer patients, the protein levels of Nrf2 and CaMKIIα in esophageal cancer tissues were analyzed by IHC. The results showed that Nrf2 was mainly expressed in the cytoplasm. A total of 58.9% of ESCC patients in our cohort showed high expression of Nrf2. CaMKIIα was mainly expressed in the nucleus. A total of 45.4% of ESCC patients showed high expression of CaMKIIα. Figure 10a shows representative IHC images of Nrf2 and CaMKIIα in ESCC tissues. Further Kaplan–Meier analysis found that the expression level of Nrf2 or CaMKIIα had no prognostic effect on patients with ESCC (Fig. 10b, c). However, the prognosis of ESCC patients with high expression of both Nrf2 and CaMKIIα was worse than that of ESCC patients with low expression of Nrf2 and high expression of CaMKIIα (Fig. 10d). All of these results indicate that the upregulation of the expression of both Nrf2 and CaMKIIα is closely related to reduced survival rates of ESCC patients.

## Discussion

Dysregulation of Nrf2 contributes to radiation resistance in ESCC, which has been confirmed in several previous studies [7–9]. However, the mechanism by which Nrf2 promotes radiation resistance in ESCC remains elusive. Here, we evaluated the effects of Nrf2 overexpression on the radiation resistance of ESCC in vitro and in vivo. Our data revealed that Nrf2-overexpressing esophageal cancer cells were more resistant to radiation than wild-type esophageal cancer cells. Consistently, the upregulation of Nrf2 reduced the radiosensitivity of ESCC in vivo. Mechanistically, we discovered that Nrf2 directly bound to the CaMKIIα promoter region to activate its transcription, which in turn resulted in increased autophagy in ESCC cells. Pretreatment with the CaMKIIα inhibitor KN-93 reversed the Nrf2-induced radiation resistance of ESCC cells. Although we confirmed that Nrf2 overexpression triggered autophagy and radiation resistance and that KN-93 treatment suppressed Nrf2-induced autophagy and radiation resistance, this evidence only indicates that there is a correlation between autophagy and Nrf2-induced radiation resistance. If Nrf2-induced radiation resistance can be reversed by the inhibition of autophagy, this finding could provide additional data to elucidate the relationship between Nrf2 and radiation resistance.

Autophagy is an evolutionarily conserved lysosomal degradation pathway that plays an important role in maintaining cell integrity [21]. Autophagic disorders are involved in many pathophysiological processes, including cancer [22–24]. Increasing evidence suggests that autophagy removes excessive reactive oxygen species (ROS) to protect cells from oxidative damage and plays a cellular protective role during radiotherapy [25, 26]. Therefore, it is expected that dysregulated autophagy might protect ESCC cells from radiation-induced cell damage, thus enhancing radiation resistance. In fact, we observed the activation of autophagy in Nrf2-overexpressing ESCC cells, accompanied by the altered expression of autophagy proteins and a reduction in radiosensitivity. In fact, crosstalk between Nrf2 and autophagy has been demonstrated in several previous studies [27–31]. It was found that p62 was a molecule that interacted with Keap1. The aberrant expression of p62 led to the suppression of Keap1-regulated Nrf2 ubiquitination and degradation [28]. Prolonged Nrf2 activation was able to promote cancer cell proliferation and treatment resistance. In addition, Paik et al. [31] found that Nrf2 could trigger the activation of autophagy in gastric epithelial cells injured by *H. pylori*, and this activation of autophagy was a key process in the adaptation to infection. In a diabetes mellitus-related cardiomyopathy (DMCMP) model, Guan et al. [29] found that high glucose mediated the activation of Nrf2 by increasing the production of ROS and further regulated the phosphoinositide 3-kinase (PI3K)/protein kinase B (AKT)/mTOR and extracellular regulated protein kinase (ERK) signaling pathways to enhance autophagy. Furthermore, Wang et al. [30] found that farrerol antagonized acetaminophen-induced hepatotoxicity by increasing the phosphorylation of adenosine 5′-monophosphate (AMP)-activated protein kinase (AMPK), AKT and PI3K and then activating autophagy via Nrf2. Although many previous studies have explored the mechanisms by which Nrf2 is involved in autophagy induction, more specific molecular regulatory mechanisms need to be elucidated.

In our previous studies, we found that CaMKIIα was a potential link between the Nrf2 signaling pathway and autophagy [17]. Therefore, in the current study, we further explored the mechanisms of Nrf2-mediated autophagy induction via CaMKIIα activation in ESCC. The Nrf2 binding sites in the CaMKIIα promoter region were screened by an online database. We confirmed the binding of Nrf2 to the ARE in the CaMKIIα promoter and the regulatory effect of Nrf2 on CaMKIIα. The suppression of CaMKIIα weakened the Nrf2-mediated induction of autophagy and the colony survival of ESCC cells treated with irradiation. In addition, Nrf2 significantly increased the radiation resistance of subcutaneous tumors by activating CaMKIIα and subsequent autophagy was also demonstrated. The results of a clinical correlation analysis suggested that the coexpression of Nrf2 and CaMKIIα was significantly correlated with poor survival in patients with ESCC. These data suggest that Nrf2 increases the transcriptional activity of CaMKIIα and then induces autophagy, resulting in resistance to radiation. Our study reveals a new mechanism by which Nrf2 triggers autophagy, which is also important for explaining the Nrf2-induced radiation resistance of ESCC.

## Conclusion

We have shown that Nrf2 induces radiation resistance in ESCC cells in vitro and in vivo by directly interacting with CaMKIIα and then triggers autophagy, which is associated with reduction in phosphorylated mTOR and p62, activation of Beclin 1, and transformation of LC3-I to LC3-II. These data indicate that Nrf2 is a promising target in the development of radiosensitization therapy for ESCC patients.

## Materials and methods

### Cell culture and transfection

Ec109 cells were purchased from BnBio Inc. (Beijing, China), and KYSE-30 cells were purchased from Sigma-Aldrich Co (St Louis, USA). Both cell lines were maintained in RPMI-1640 medium (Gibco, CA, USA) supplemented with penicillin/streptomycin (1%) and heat-inactivated FBS (10%). The cells were cultured at 37 °C in an atmosphere of 5% CO_2_ and 95% room air.

For the overexpression of Nrf2, Ec109 and KYSE-30 cells were seeded in six-well plates. Once the cells reached 30–40% confluence, the corresponding amount of lentivirus (HanBio, Shanghai, China) and polybrene transfection reagent were added. After 72 h of transduction, the positively transduced cells were selected with puromycin for another 72 h. For Nrf2-siRNA transfection, the predesigned siRNA targeting the Nrf2 gene and the negative control siRNA were purchased from Invitrogen (Massachusetts, USA). The Nrf2 siRNA or negative control siRNA was transfected with Lipofectamine RNAi-Max transfection reagent (Invitrogen, USA). The transfection effect was subsequently confirmed.

### qRT-PCR

Total RNA was extracted from the ESCC cell lines by TRIzol (Ambion, USA). For cDNA synthesis, 500 ng of total RNA was reverse transcribed in a 10-μl reaction volume using the PrimeScriptTM RT Master Mix (Perfect Real Time) kit (Takara, Japan). After cDNA amplification, the resulting cDNA was subjected to quantitative PCR using SYBR^®^Premix Ex Taq TM II (Tli RNaseH Plus) (Takara, Japan). β-actin was selected as the housekeeping gene. The expression level of each gene was calculated by the 2^−ΔΔCt^ method. The following primers were used for qRT-PCR: Nrf2, 5′-ACACGGTCCACAGCTCATC-3′ and 5′-TGTCAATCAAATCCATGTCCTG-3′; NQO1 ARE, 5′-CCCTTTTAGCCTTGGCACGAAA-3′ and 5′-TGCACCCAGGGAAGTGTGTTGTAT-3′; CaMKIIα ARE, 5′-CAGGGCAAAGAGGAGCAGG-3′ and 5′-GATTCTCCTCCACGTCACCGC-3′; and β-actin, 5′-CTCCATCCTGGCCTCGCTGT-3′ and 5′-GCTGTCACCTTCACCGTTCC-3′.

### Western blotting

Cells were lysed with radioimmunoprecipitation assay (RIPA) buffer (50 mmol HEPES at pH 7.5, 150 mmol NaCl, 10% glycerol, 1.5 mmol MgCl_2_, 1% Triton-X 100, 1 mmol ethylenediamine tetraacetic acid at pH 8.0, 10 mmol sodium pyrophosphate, 10 mmol sodium fluoride) containing protease inhibitor and phosphatase inhibitor cocktails. After thermal denaturation at 95 °C for 5 min, the protein samples were separated by 10% SDS-PAGE. After transfer, the membranes were then incubated with 5% skim milk to block the nonspecific binding of antibodies. Subsequently, the membranes were incubated with primary antibodies against Nrf2 (Abcam, UK), TBP (Bioss, China), phosphorylated CaMKIIα (Abcam, UK), CaMKIIα (Abcam, UK), phosphorylated mTOR (Affinity, USA), Beclin 1 (CST, USA), p62 (CST, USA), LC3 I/II (CST, USA), and GAPDH (Bioss, China) overnight at 4 °C. After incubation with the primary antibodies, horseradish peroxidase (HRP)-conjugated goat anti-mouse/rabbit secondary antibodies (ZSGB-bio, China) were incubated for an additional 1 h. Finally, the membranes were visualized with an enhanced chemiluminescence reagent. The relative levels of protein expression were normalized against that of GAPDH as the internal control.

### CCK-8 assay

Cell viability was examined using the CCK-8 kit (Beyotime, China). Briefly, ESCC cells were seeded in ninety-six-well plates. Once the cells reached 40% confluence, they were irradiated with 4 Gy X-rays (Varian linear accelerator, USA, at a dose rate of 300 MU/min). Next, the CCK-8 solution was added and incubated for 2 h. The absorption value was measured at 450 nm with a microplate reader.

### Colony formation assay

Cells were treated with increasing doses of irradiation (0–6 Gy) using the Varian linear accelerator (dose rate of 300 MU/min). After irradiation, the cells were then cultured for 14 days. Finally, the cells were fixed with methanol, stained with crystal violet and counted. The survival curve was generated according to the linear-quadratic model.

### Flow cytometry analysis

Wild-type and Nrf2-overexpressing ESCC cells were incubated in phenol red-free culture medium (Invitrogen, USA) and diluted Cyto-ID^®^ Green stain solution (Enzo, USA). After incubation, the samples were analyzed by flow cytometry.

### Confocal fluorescence microscopy

Cells were seeded on coverslips. Once the cells reached 40% confluence, they were stained with the Cyto-ID^®^ reagent and observed using a Leica laser scanning confocal microscope. The fluorescence intensity of the cells, representing the occurrence of autophagy, was calculated by ImageJ (NIH, USA).

### ChIP–qPCR assay

The ChIP-qPCR assay was carried out with the One-Day Chromatin Immunoprecipitation Kit (Millipore, USA). Ec109 cells were crosslinked with 1% formaldehyde and then sequentially lysed with lysis buffer 1 (50 mM HEPES–KOH pH 7.5; 140 mM NaCl; 1 mM EDTA; 10% glycerol; 0.5% NP-40; 0.25% Triton X-100) and lysis buffer 2 (10 mM Tris–HCl pH 8.0; 100 mM NaCl; 1 mM EDTA, pH 8.0; 0.1% Na-deoxycholate, protease inhibitors). To obtain DNA fragments, the lysates were sonicated, and the DNA was sheared into 300–500-bp fragments. Then, 250 μl chromatin-DNA mixture and 50 μl magnetic beads (Bangs Laboratories, USA) were used for immunoprecipitation with an anti-Nrf2 antibody. The cross-linking was reversed, and the proteins were eliminated by incubation with proteinase K. The purified DNA was analyzed by qPCR.

### Animal study

A total of 1 × 10^6^ wild-type or Nrf2-overexpressing Ec109 cells were subcutaneously injected into nude mice (CAS, Beijing, China). The tumors were allowed to grow for approximately 10 days after injection, when the volume reached nearly 150 mm^3^. Subsequently, the mice were randomized and assigned to four groups (n = 6 per group): (1) Control, (2) Control + irradiation, (3) Nrf2 overexpression, and (4) Nrf2 overexpression + irradiation. The tumors were irradiated with 6 Gy X-ray. After 4 weeks of follow-up, the mice were sacrificed, and the tumors were removed for further analysis. A formula, volume = 0.5 × (length × width^2^), was used for the calculation of tumor volume. The animal study was carried out in accordance with the guidelines of the International Association for Experimental Animal Care Assessment and Certification.

### Patients and tissue samples

Forty-four patients with ESCC who underwent esophagectomy with lymph node dissection at The General Hospital of Ningxia Medical University were enrolled in the study. A total of 49.5% of the patients received adjuvant radiotherapy. However, none of the enrolled patients received new adjuvant radiotherapy or chemotherapy. Tumor histology specimens were collected from each patient. The clinicopathological features of ESCC patients with high expression of CaMKIIα are listed in Additional file 1: Table S1. OS was defined as the time from the pathological diagnosis of ESCC until death or last follow-up. The use of all the human ESCC tissues and clinicopathological data was approved by the Ethics Committee of the General Hospital of Ningxia Medical University (2015-088).

### Immunohistochemistry analysis

The specimens of either the xenograft model or the ESCC patients were stained by IHC. Antigen retrieval was performed by high pressure. Then, a 3% hydrogen peroxide solution was used to reduce the activity of the endogenous peroxidase. Subsequently, the slides were incubated with the indicated primary antibodies at 4 °C overnight. Antigen–antibody binding was visualized with 3,3′-diaminobenzidine (DAB). Finally, the slides were assessed by pathologists and evaluated using a semiquantitative method as previously described [32]. The primary antibodies included Nrf2 (Abcam, UK), CaMKIIα (Bioss, China), LC3-II (Bioss, China), and Ki-67 (Bioss, China).

### Statistical analysis

Student’s t test or analysis of variance (ANOVA) was carried out to evaluate the differences among the groups. The Chi square test was used to determine the association of categorical variables. OS curves were drawn by the Kaplan–Meier method, and the differences among subgroups were evaluated by the log-rank test. SPSS 22.0 (Armonk, USA) and GraphPad Prism 8.0 software (San Diego, USA) were used in the analysis. When *P *< 0.05, the data were considered to be statistically significant.

## Supplementary information

**Additional file 1: Table S1.** Clinicopathological features of ESCC patients with high expression of CaMKIIα.

## Data Availability

All data generated or analyzed during this study are included in this published article.

## Abbreviations

ARE: Antioxidant response element
CaMKII: Ca2 +/calmodulin-dependent protein kinase II
CCK-8: Cell counting kit-8
ChIP–qPCR: Chromatin immunoprecipitation (ChIP) –qPCR
CRT: Chemoradiotherapy
DMCMP: Diabetes mellitus-related cardiomyopathy
eEF2K: Eukaryotic extension factor 2 kinase
ERK: Extracellular regulated protein kinases
ESCC: Esophageal squamous cell carcinoma
HMGB1: High mobility group box 1
HO-1: Heme oxygenase-1
IHC: Immunohistochemistry
Keap1: Kelch-like ECH related protein 1
LKB1: Liver kinase B1
NQO1: NAD(P)H dehydrogenase quinones 1
Nrf2: NF-E2 related factor 2
OS: Overall survival
qRT-PCR: Quantitative real-time polymerase chain reaction
ROS: Reactive oxygen species
