# Supplementary material for: Nrf2 promotes esophageal squamous cell carcinoma (ESCC) resistance to radiotherapy through the CaMKIIα-associated activation of autophagy
Source: Cell Biosci. 2020 Jul 30;10:90. doi: 10.1186/s13578-020-00456-6 (PMC7392680; doi:10.1186/s13578-020-00456-6)
Supplement: Supplementary file 1 — Additional file 1: Table S1. Clinicopathological features of ESCC patients with high expression of CaMKIIα. [file 13578_2020_456_MOESM1_ESM.docx]

Table S1 Clinicopathological features of ESCC patients with high expression of CaMKIIα.

| Clinicopathologic characteristics | N | Nrf2-/CaMKIIα+ | Nrf2+/CaMKIIα+ | P value |
| --- | --- | --- | --- | --- |
|  |  |  |  |  |
| Gender |  |  |  |  |
| Male | 17 | 10 | 7 |  |
| Female | 3 | 2 | 1 | 1.000 |
| Age (years) |  |  |  |  |
| <60 | 6 | 4 | 2 |  |
| ≥60 | 14 | 8 | 6 | 1.000 |
| KPS |  |  |  |  |
| >80% | 10 | 5 | 5 |  |
| ≤80% | 10 | 7 | 3 | 0.650 |
| Weight loss (kg) |  |  |  |  |
| <5 | 16 | 10 | 6 |  |
| ≥5 | 4 | 2 | 2 | 1.000 |
| Drinking |  |  |  |  |
| Yes | 5 | 2 | 3 |  |
| No | 15 | 10 | 5 | 0.347 |
| Smoking |  |  |  |  |
| Yes | 12 | 5 | 7 |  |
| No | 8 | 7 | 1 | 0.070 |
| Histological differentiation |  |  |  |  |
| Well and moderate | 14 | 11 | 3 |  |
| Poor | 6 | 1 | 5 | 0.018 |
| Tumor length (cm) |  |  |  |  |
| <3 | 4 | 3 | 1 |  |
| ≥3 | 16 | 9 | 7 | 0.619 |
| Infiltration depth |  |  |  |  |
| T1 and T2 | 5 | 4 | 1 |  |
| T3 and T4 | 15 | 8 | 7 | 0.603 |
| Lymph node status |  |  |  |  |
| N0 | 13 | 10 | 3 |  |
| N1 | 7 | 2 | 5 | 0.062 |
| pTNM staging |  |  |  |  |
| I and II | 13 | 10 | 3 |  |
| III | 7 | 2 | 5 | 0.062 |
